# Skill development practice-related challenges, and associated factors among nursing students

**DOI:** 10.1186/s12912-024-02115-4

**Published:** 2024-06-25

**Authors:** Bizuayehu Atinafu Ataro, Almaz Addisie, Temesgen Geta Hardido, Getachew Nigussie Bolado, Dawit Simeon Bilate, Yakob Lencha Leka, Beskut Bezabih

**Affiliations:** 1 Wolaita Sodo University, Wolaita Sodo, Ethiopia; 2 Addis Ababa University, College of Medicine and Health Sciences, Addis Ababa, Ethiopia; 3 Dawro Tarcha Teaching Hospital, Dawro Tercha, Ethiopia

**Keywords:** Skill development practice, Challenges, Associated factors, Nursing student

## Abstract

**Background:**

Nurse students reportedly face lots of challenges during skill development practice in health institutions. However, the prevalence of challenges and factors associated aren’t well understood yet.

**Objective:**

The objective of the study was to identify the challenges experienced by nursing students in health institutions during skill development practice.

**Method:**

A cross-sectional study was employed on the participants selected using a simple random sampling technique. The data was cleaned using Epi-data and exported to SPSS for analysis. Logistic regression analysis and correlation analysis were carried out to identify the associated factors.

**Result:**

The participants are more challenged by instructor factors (43.6%) and facility factors (40%). The prevalence of the challenge was 16.9%. Substance use and learning institutions are found to be independent predictors. A negative correlation was identified between the total challenge score and the overall competency score.

**Conclusion:**

The determined nursing students’ challenges are strong enough to affect the quality of education; therefore, it is essential to plan and improve the students’ integrated supportive supervision.

## Background

Nursing education contains theoretical and practical aspects. A large portion of nursing education is undertaken at health institutions. Clinical skill development institutions play an essential role in achieving professional competence, as nursing is a practical-based discipline. The clinical practice environment can determine nursing students’ choice or rejection of a nursing field as a profession. The teaching and learning process conducted at a clinical exercise facility is affected by lots of factors even though it paves the way for nursing students to gain mental, psychological, and psychomotor skills that are necessary for patient care [1–9].

The clinical learning environment includes the clinical setting, clinical staff, patients, and tutors. Challenges around the clinical environment affect the quality of nursing education and influence the achievement of learning outcomes; hence, there should be a plan to select the best clinical learning environment, which is difficult to achieve in developing nations [1, 2].

An internship site is a potential source of anxiety, disappointment, and disgust; therefore, the link between the instructors, staff nurses, and patients is very essential to form the student’s clinical training, to cope, and to reduce the real shock [1]. As the students are required to equip themselves with the expected skills such as independence, critical thinking, communication, time management, responsibility, accountability, and clinical judgment via clinical practice, the quality of the practicum setting is vital and fine to realize the demanded product by averting the upcoming institutional shock [3]. A standardized clinical practice institution has a positive effect on the student’s professional growth, whereas a suboptimal clinical practice environment could retard their professional development process [10, 11].

The academic experience developed by poor clinical instruction exposes the students to performance alteration in the clinical area, which in turn negatively affects their skill development at health institutions [11]. Identifying challenges encountered by nursing students during clinical practice could help the stakeholders solve the identified problems, which contributes to professional endurance [2]. Whereas reluctance to recognize the challenges could bump the teaching and learning process productivity at a health facility [12].

The students’ ineffective contact with the clinical learning environment reportedly resulted in a significant surge in the academic failure rate. The outnumbered nursing students are forced to leave their profession as a result of the many challenges experienced during skill development practice [13–16].

The nursing students’ inadequate skills lead to anxiety in real work. In significant numbers of the highest institutions, fresh graduates have plenty of theoretical knowledge, but they aren’t competent enough in the skill aspect. To boost the quality of health system function with adequate numbers of skilled, interested, and reinforced nurses who show good work ethics at all times, the possible constraints around skill development sites should be identified and solved [11, 14].

The clinical area where nurses acquire clinical skills in sub-Saharan Africa is challenged by individual factors (student and nurse tutor factors), socioeconomic factors, and the hospital environment [16–18]. A study done in Ghana showed that nursing students failed to apply theory to practice because of the absence of a strong supportive supervision system in clinical settings [6].

Some students have an unfavorable attitude toward clinical practice, and the majority of them reported that a late approach to clinical practice makes them not adequately prepared for clinical practice; therefore, more supportive and relevant interventions should be implemented based on the identified challenges to help the students achieve a higher level of skill development. Furthermore, determining the challenges being bumped during professional skill development could help to work on the gap, advance training, and boost the quality of clinical practice [15, 19–22]. Therefore, the current study was carried out to identify the challenges in health institutions faced by nursing students.

### Study objectives

- To determine the challenges experienced by nursing students during skill development practice in health institutions.
- To identify the factors associated with challenges faced by nursing students during skill development practice in health institutions.

## Materials and methods

### Research design and setting

A cross-sectional study was carried out at the highest institutions of Addis Ababa City, which is the capital city of Ethiopia and the headquarters of the African Union. The study was conducted from February 15 to April 30, 2022.

### Participants and sample size calculation

All third- and fourth-year undergraduate nursing students with over one year of clinical practice experience from both public and private higher institutions who are available during a study period were considered as a study population and included in the study.

The sample size was determined by a single population proportion formula. A simple random sampling technique was used for the selection of study subjects. N was 373, and P was 25.2% from previous research [21]. Based on this formula *n* = 290. As the study population was less than 10,000 finite populations, a correction formula was applied to get$163$, and after adding a 5% non-responsive rate, the final sample size required for this study was 172 subjects.

**Fig. 1:**
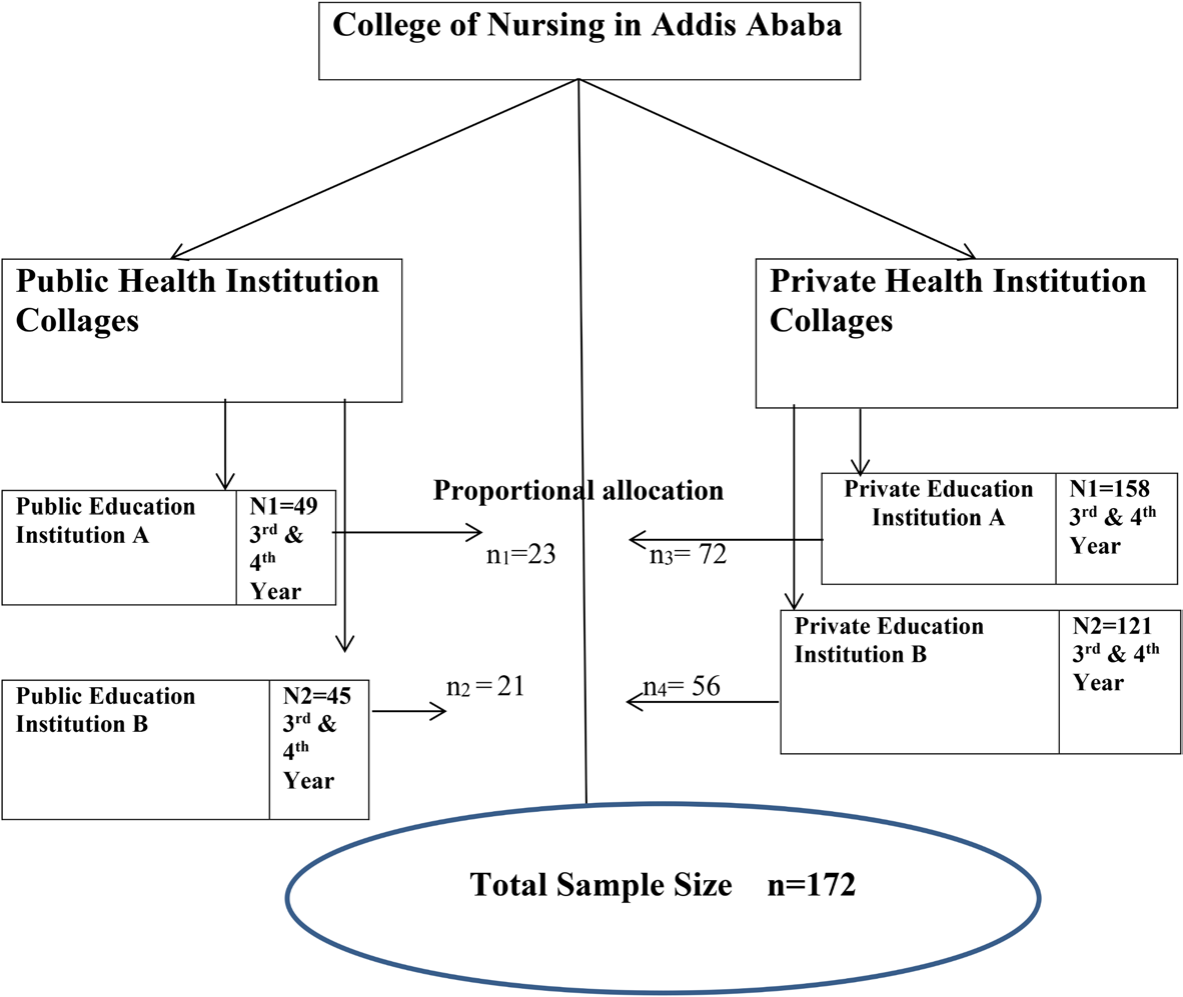
Sampling procedure

The final sample size was allocated to the selected institutions by proportion [22] (Fig. 1).

### Variables

#### Dependent variable

Challenges faced during skill development practice.

#### Independent variables

Socio-demographic characteristics - age, sex, ethnic group, marital status, religion, and others.

Possible factors affecting clinical setting practice - individual student nurse factors, staff nurses factors, instructor factors, management factors, facilities/clinical education factors, and clinical practice competence-related factors.

### Operational definitions

Challenge: score ranges from 40 to 200.

More challenged or higher challenge score- a median score of 120 and above.

The rest were categorized as having minimal or low challenge.

With one item it was 3, and with 40 items it was 120.

### Data collection and tool

The data was collected by trained data collectors. The data was collected by an English-version tool adapted from a related study and amended after the pilot test based on the prevailing context of nursing schools in Ethiopia to make it fit the study population [21, 23]. After the pilot test, the content validity was tested, the tool had good construct validity, and Cronbach’s alpha was 0.831. The entailed changes to the tool include: ambiguity and interpretation-related issues were identified and resolved; errors were identified and fixed; the response rate was identified; discrepancies were detected and managed; and the appropriateness of the allotted time was determined and revised. The instrument had three sections. Section one: sociodemographic variables include age, sex, institution name, marital status, and others. Section two: a closed-ended item that assesses a challenge in a clinical setting. Section three: six closed-ended items for competency assessment. A tool had a 5-point Likert scale to represent the challenges in a clinical setting for a topic scored on a 5-point scale.

### Data analysis

Epidata and Statistical Package for Social Science (SPSS) software version 26 were used for this study to clean and analyze the data, respectively. Logistic regression analysis and correlation analysis were carried out to identify the associated factors, and significance was declared at a p-value of < 0.05.

## Results

### Socio-demographic characteristics of the study participants

A total of 172 nursing students participated in the study, with a response rate of 100%. Of the 172 study participants, 123 (71.5%) were female, and 133 (77.3%) of them were in the age group of 18–25 years. 108 (62.8%) were Orthodox religious followers, and 69 (40.1%) were from St. Mary Health Science College (Table 1).

**Table 1:** Socio-demographic characteristics of the participants

Variables	Frequency	Percent
**Sex**		
Female	123	71.5
Male	49	28.5
**Age group**		
18–25	133	77.3
26–34	39	22.7
**Religion**		
Orthodox	108	62.8
Protestant	31	18.0
Muslim	22	12.8
Catholic	11	6.4
**Current Marital status**		
Single	143	83.1
Married	29	16.9
**Learning institution**		
Public Education Institution A	23	13.4
Public Education Institution B	22	12.8
Private Education Institution B	58	33.7
Private Education Institution A	69	40.1
**Entrance year**		
Third	82	47.7
Fourth	90	52.3
Residence (*n* = 171)		
Dormitory	17	9.9
Rental Home	104	60.8
Other	50	29.2
**Substance use**		
Yes	18	10.5
No	144	83.7

### Prevalence of challenge

Concerning the management domain, more challenges are reported on the resource or equipment aspect. Furthermore, 12.2% of the participants reported failure in communication between students and tutors. 11% of the participants reported insufficient preparation skills before the internship, and 51.2% of the participants indicated that they were challenged by resource or equipment limitations. Participants were most challenged by instructor factors (43.6%). 9.3% of the participants mentioned that they feel discriminated against when not allowed to participate in patient rounding with students of medicine and reported the presence of discrimination between them and other health science students.

**Fig. 2:**
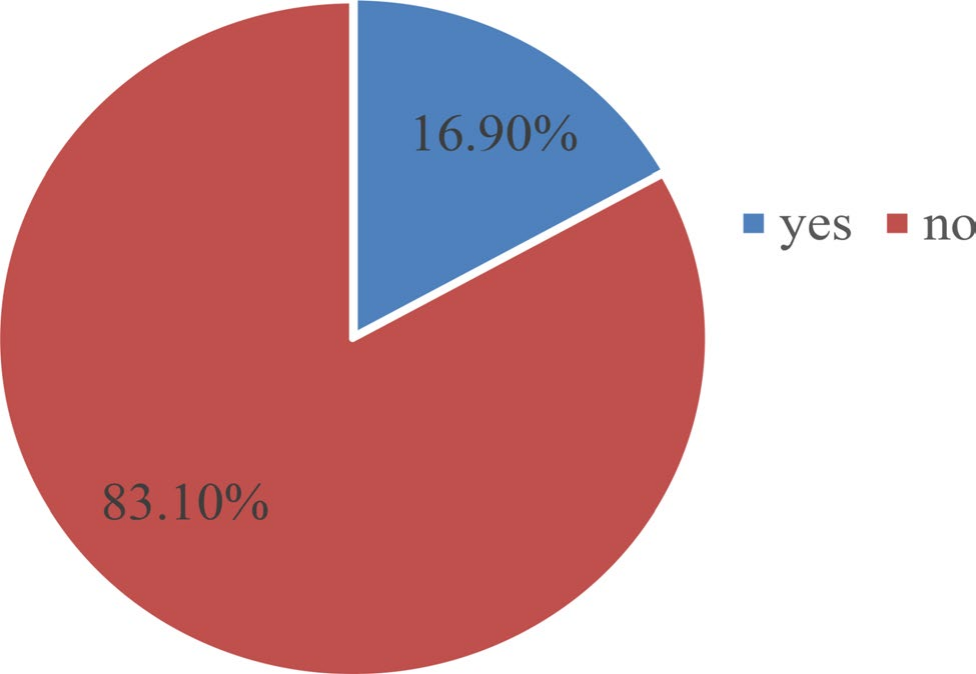
Prevalence of Challenges among the participants

The prevalence of challenges among nursing students was found to be 16.9% (Fig. 2).

### Factors associated with the challenge of nursing students

Religion, residence, entrance year, learning institution, and substance use were found to have an association with challenges in the bivariate analysis. However, after controlling for the effect of confounding factors in multivariable analysis, substance use and learning institutions were found to have a significant association.

A statistically significant negative moderate correlation was identified between the total challenge score and the overall competency score. This implies that as the students’ challenge score increases, their competency score decreases. This finding is supported by bivariate logistic regression: for each additional score in the total challenge score, the odds that the nursing students will have a good competency decrease by about 3% (Table 2).

**Table 2:** Factors associated with the challenge of nursing students

Explanatory variables	Challenge status		COR 95% CI	AOR 95% CI	*P*-Value
	Yes	No			
**Learning institution**					
Public Education Institution A	9	14	1.82 (0.67, 4.93)	0.18 (0.01, 3.62)	0.266
Public Education Institution B	1	21	0.13 (0.02, 1.08)	0.10 (0.01, 0.98)	0.048*
Private Education Institution B	1	57	0.05 (0.01, 0.39)	0.03 (0.003, 0.29)	0.002*
Private Education Institution A	18	51	1	1	
**Residence**					
Dorm	9	8	7.54 (2.61, 21.80)	15.91(0.81, 312.45)	0.069
Non-dorm	20	134	1	1	
**Substance use**					
Yes	7	11	3.53 (1.23, 10.09)	8.25 (1.74, 39.04)	0.008*
No	22	122	1	1	
**Entrance year**					
Third	17	65	1.70 (0.76, 3.82)	1.18 (0.44, 3.12)	0.745
Fourth	12	78	1	1	

*Statistically significant at p-value < 0.05; 1 is the Odds ratio for the reference category

## Discussion

The prevalence of challenges among nursing students was found to be 16.9%, which is lower than a study conducted in the Amhara region, northern Ethiopia (25.2%), and the University of Gondar, Northwest Ethiopia (48.7%) [16, 24]. The possible reason for the variation is the difference in the characteristics of the study participants. Some studies included only public learning institutions, while others included only private institutions, whereas this study included both public and private higher education institutions.

The odds of having a challenge for study participants who were substance users were 8.25 times higher than those who were not substance users. The possible justification for this association might be that substance users are less likely to comply with the standards of the teaching and learning process, which might have the potential to develop a negative attitude toward those who teach them, such as instructors and nurse staff.

The percent odds of facing challenges for those who were learning in Public Education Institution B were 90% lower than those learning in Private Education Institution A. Similarly, the percent odds of having a challenge for the participants from Private Education Institution B were 97% less than those of the participants from Private Education Institution A. This implies that these two learning institutions, namely Public Education Institution B and Private Education Institution B, are using a relatively good practical education system for their students. In a study done in Iran, Benha Public Hospital had the highest mean score of challenge (65.3%) among private hospitals [17].

For action to take place in practical areas to resolve problems, Wambui et al.’s study [13] revealed that proper treatment and communication with students an important items for nursing teachers to be role models for students. That remark agrees with the findings of this study as 12.2% of the students in this study lack proper communication with their instructors.

9.3% of the participants mentioned that they feel discriminated against when not allowed participation in patient rounding with students of medicine and reported the presence of discrimination between them and other health science students; the finding was consistent with a study done in Iran [17]. The students’ lack of skill as a result of inadequate preparation before arriving at the clinical environment creates problems for them and nursing teachers (25–26), those findings are supported by this study’s finding as about 11% of the participants didn’t have sufficient preparation before the internship. Concerning the management domain, 51.2% of the study participants indicated that they are challenged by resource or equipment limitations. This showed that the participants of this study were more challenged than the participants of a study conducted in Northwest Ethiopia (22.9% of the participants were outspoken about the shortage of equipment that affected their skill development accomplishment) [24].

Generally, the participants are more challenged by instructor factors (43.6%) which corroborates the findings of other studies [27]. Hence, nurse instructors should undertake strong supportive supervision and should have effective communication with their students.

### Limitations of the study

The study was undertaken via an institutional-based comparative cross-sectional study design using a self-administered English version of a questionnaire adapted from a related study, and amended after the pretest; hence the study didn’t address the issue of casual relationships. Therefore, the authors recommend that researchers consider a longitudinal study and a mixed approach while thinking about conducting further studies on related titles.

## Conclusion

The prevalence of challenges nursing students face in their practical education was found to be reasonable. There was a moderately negative correlation between the students’ challenge score and their competency score, which indicates the negative effect of the student’s challenge on their competency level. Therefore, statistically significant factors should be considered in the effort made to reduce the students’ challenge level and improve the quality of practical education.

The instructors and clinical facilitators are better able to assist the students in linking theory with practice successfully, as a higher percentage of the student nurses are being challenged by instructor-related factors. Working on the reduction of substance use among the students is demanded to reduce the challenge resulting from substance use and to improve the competency of the students as well.

Generally, the results of this study could have a significant contribution to nursing practice and the development of nursing skills as it has identified major challenges encountered by nursing students during skill development practice at health institutions. So that the stakeholders and the responsible bodies can work on the identified major challenges and associated factors to improve the quality of education, particularly in the practicum domain.

## Data Availability

All information and materials described in the research will be freely available to any researchers wishing to use them for non-commercial purposes without breaching participant confidentiality. The datasets used during the current study is not publicly available in order to maintain data security but is available from corresponding author on reasonable request.

## Abbreviations

AAU: Addis Ababa University
BSc: Bachelor of Science
CLE: Clinical learning environment
ETB: Ethiopian Birr
FGD: Focus Group Discussion
GC: Gregorian Calendar
HWs: Health Workers
IQR: Interquartile Range
IRB: Institutional Review Board
OR: Operation Room
